# Is there a semantic system for abstract words?

**DOI:** 10.3389/fnhum.2013.00175

**Published:** 2013-05-08

**Authors:** Tim Shallice, Richard P. Cooper

**Affiliations:** ^1^Cognitive Neuroscience Sector, SISSATrieste, Italy; ^2^Institute of Cognitive Neuroscience, University College LondonLondon, UK; ^3^Department of Psychological Sciences, Birkbeck, University of LondonLondon, UK

**Keywords:** semantics, concepts, abstract words, embodied cognition, left ventrolateral prefrontal cortex

## Abstract

Two views on the semantics of concrete words are that their core mental representations are feature-based or are reconstructions of sensory experience. We argue that neither of these approaches is capable of representing the semantics of abstract words, which involve the representation of possibly hypothetical physical and mental states, the binding of entities within a structure, and the possible use of embedding (or recursion) in such structures. Brain based evidence in the form of dissociations between deficits related to concrete and abstract semantics corroborates the hypothesis. Neuroimaging evidence suggests that left lateral inferior frontal cortex supports those processes responsible for the representation of abstract words.

## Introduction

Probably the best known theorizing on the organization of the semantic system within the embodied or grounded cognition approach is that of Barsalou (2008). While the theory has not been implemented, it would appear that the systems involved in the representation and use of abstract concepts, in particular the perceptual system and those responsible for frames and simulations, are the same as those required for the representation and use of concrete concepts (but see Adams and Campbell, 1999; Mitchell and Clement, 1999; Ohlsson, 1999). In this paper we will discuss neuropsychological and functional imaging evidence which suggests that the representation of abstract concepts, in fact, involves a system additional to those involved in the semantic representation of concrete concepts. We will then discuss what computationally could give rise to this separability between abstract and concrete words within the functional architecture of the semantic system.

As far as neuropsychological evidence is concerned, we will specifically discuss two syndromes—deep dyslexia and the selective preservation of abstract concepts in the so-called reversed concreteness effect found in some semantic dementia and herpes encephalitis patients. The first of these functional syndromes—deep dyslexia—appears to provide evidence for at least partial separability of the semantic representations of concrete and abstract words. The prototypic characteristic of the deep dyslexic patient, and generally why their reading difficulty was analyzed, is the making of semantic errors when reading aloud. However, there are also a variety of other characteristics in their reading that such patients have in common (Coltheart et al., 1987; Plaut and Shallice, 1993). One is that the patients are much more able to read aloud words with a concrete, or better an imageable, meaning than those with an abstract meaning (Shallice and Warrington, 1975; Coltheart et al., 1987). *Face* can be read but not *faith*. Moreover, for many of these patients the difference is very large. Thus Shallice (1988) considered the performance of the first four deep dyslexics whose reading was analyzed in detail; the smallest difference between the reading of concrete and abstract words was in patient GR of Marshall and Newcombe (1966) who read aloud 50% of the former but only 10% of the latter.

Since it is standardly accepted that the phonological route or routes for reading are inoperative in these patients, it would seem straightforward to produce an explanation for their inability to read abstract words which is based on the assumption that there are different systems for holding the semantic representations of abstract and imageable/concrete words. Thus, if one assumes that there is an at least partial separability between the semantic systems holding representations of imageable and abstract words, then it is simple to assume that in this functional syndrome the latter subsystem is no longer directly accessible from a visual word-form system, while the former subsystem is. This, indeed, was the explanation for this aspect of the deep dyslexia functional syndrome given by Morton and Patterson (1980).

There are, however, two reasons to be cautious about this interpretation. The first is that the abstract-to-concrete difference is only one of the many characteristics of deep dyslexia, and this type of explanation of the functional syndrome as a whole is not very economical; thus Morton and Patterson (1980) require five separate functional impairments to explain all aspects of the functional syndrome. Secondly, it is possible to provide an explanation of the superiority of concrete over abstract word reading assuming that exactly the same set of systems are involved in reading for both types of word, but that the semantic representations of abstract words are in some sense quantitatively weaker than those of concrete words. Thus in the connectionist model of Plaut and Shallice (1993), the attractor structure leads to an abstract-concrete difference; the reduced number of features of abstract words by comparison with concrete words make the processes involved in the reading of abstract words less able to support a clean-up process than can the richer semantic representations of concrete words.

A second possible type of explanation, which is also compatible with abstract and concrete words being represented semantically in the same system is given by Newton and Barry (1997). Deep dyslexia can be present in either an input, central or output form, depending where the impairment lies in the semantic route for reading, it being assumed that the phonological route or routes are inoperative (Shallice and Warrington, 1980). Newton and Barry studied a patient, LW, with an output form of deep dyslexia—his comprehension of the written word was intact—but who showed a large effect of concreteness on his ability to read aloud (highly concrete 48%; abstract 8%). These authors hold that, when using only the semantic route for reading, the contrast in performance between concrete and abstract words in LW could be due to the greater difficulty that the abstract words produce for the lexicalization process by which an output phonological word-form or lemma is produced from a semantic representation. Indeed, Barnard et al. (1982) showed that it was much easier for normal subjects to name from definition concrete words, such as *barrel* (77% correct), than abstract words, such as *betray* (23% correct). Newton and Barry plausibly argue that concrete words have a higher degree of specificity in the lexicalization process than abstract words like *idea*, for which they claim “there will be a great deal of spreading activation to many… related concepts” (p. 502). More generally it has been argued that accessing abstract and concrete words involves the same semantic system but that access also requires a network of prior knowledge, and abstract words are more heavily dependent on this network (Schwanenflugel, 1991). So loss of access to the network could give rise to the deep dyslexic pattern of concrete word superiority.

These two examples of explanations of better performance on concrete than abstract words both depend on concrete words being higher on some quantitative dimension—number of features or degree of specificity—than abstract words. Moreover, intuitively there are no apparent processes where on a relevant dimension abstract words would be easier to operate on than concrete words. There is one model—that of Plaut and Shallice (1993)—where higher performance on abstract than concrete words can occur, but this requires a rather specific set of assumptions. It is clear that if a much stronger case for the separability of systems underlying concrete and abstract semantic representations in the relative preservation of abstract concepts compared with concrete ones can be found, then an explanation in terms of their different placings on an underlying continuous dimension is much more difficult to produce.

## The reversed concreteness effect

The first patient to be described with the reversed concreteness effect—the better processing of abstract rather than concrete (or imageable) words—was AB of Warrington (1975). AB was asked to provide the meaning of a set of abstract and concrete words. He was rated as producing an appropriate description of the meanings of 85% of the abstract words but only 24% for the concrete ones. Thus he described a *pact* as “friendly agreement” and *arbiter* as “He is a man who tries to arbitrate. Produce a peaceful solution.” But to *hay* and *needle*, he said he had forgotten the meaning. AB suffered from what would now be known as semantic dementia. Later patients showing the reversed concreteness effect have also been described with semantic dementia (see e.g., Breedin et al., 1994; Cipolotti and Warrington, 1995; Bonner et al., 2009; Macoir, 2009; Papagno et al., 2009a).

A second aetiology in which the reversed concreteness effect was obtained is herpes simplex encephalitis. Warrington and Shallice (1984) described patient SBY who was 94% correct at giving the meaning of abstract words, but only 50% correct at giving the meaning of concrete words. Further patients with the reversed concreteness effect following herpes simplex encephalitis have since been described by Sirigu et al. (1991) and Mattioli (2008).

One critical property of these two aetiologies—semantic dementia and herpes simplex encephalitis—is that they are both conditions generally giving rise to so-called semantic degradation rather than semantic access difficulties, when they affect the semantic system (see Warrington and Shallice, 1979, 1984; Warrington and Cipolotti, 1996). In particular, there tends to be high consistency across sessions in whether or not a patient with one or other of these two conditions knows the meaning of a word (Warrington and Shallice, 1984; Warrington and Cipolotti, 1996). Thus, it is argued that the deficit is of the semantic representations themselves rather than in accessing or retrieving them.

A second critical property is that both have primary lesion sites in the anterior temporal lobes. In semantic dementia the critical lesion site is thought to be in the inferior parts of the anterior temporal cortex (Mummery et al., 2000; Mion et al., 2010) and this would be the site of any hypothetical semantic “hub” as on the theory of Rogers et al. (2004). There are some suggestions that the critical lesion site is more lateral than medial (e.g., Binney et al., 2010), but this is less clear in other studies (e.g., Mion et al., 2010). For the semantic deficits characteristic of herpes simplex encephalitis, where category specificity within the semantics of concrete entities is more typical (Capitani et al., 2003), the critical lesion site is again inferior anterior temporal cortex, but in this case potentially more medial than lateral (e.g., Tyler et al., 2004).

The reversed concreteness effect, as discussed so far, has been demonstrated only in individual patients selected for study because they show this characteristic. However, recently there have been criticisms of drawing inferences from individual case studies to the organization of the normal cognitive system, in particular with respect to category specificity, of which the reversed concreteness effects is one example (Laws, 2005; Laws and Sartori, 2005). It is possible that patients showing a reversed concreteness effect are premorbidly biased, with respect to the average of the population, in how well abstract concepts are represented by comparison with concrete ones (Hoffman and Lambon Ralph, 2011). This makes studies using a case series methodology, in which patients are selected because of their aetiology and not because of their behavioral characteristics, particularly important (Schwartz and Dell, 2010; Shallice and Buiatti, 2011). Three research studies have been carried out. One study, that of Yi et al. (2007), was only concerned with the comprehension of verbs, which in general we will not deal with in this paper. Unfortunately the results of the other two point in opposite directions.

In the first of the other two studies, Hoffman and Lambon Ralph (2011) recruited seven patients with a diagnosis of semantic dementia and gave them all seven tests, each of which compared comprehension of abstract and concrete words. Two involved only verbs. The other five involved synonym judgments, description-to-word matching, picture-to-word matching and word-to-related word matching. No patient performed significantly better on the abstract words on any test. Three of the patients performed at a very similar level on the concrete and abstract words, but three performed significantly better overall on the concrete words, if the two verb processing tasks are included. Hoffman and Lambon Ralph draw the conclusion that the reversed concreteness effect is an artifact of the selection of premorbidly atypical patients. There is, however, a major problem with their study. There is no control group. As discussed above most people in most tasks find abstract words more difficult to process than concrete ones. We do not know whether the pattern of performance shown by the semantic dementia patients in this case series produced the typical level of difference between abstract and concrete words that an impaired general-purpose semantic system would show or whether the relative difference between the two types of words was in fact less than that normally found, especially for the three patients who showed very similar levels of performance between the two types of word.

By contrast, the study of Loiselle et al. (2012) did have a control group; in fact it had two. It compared 7 patients having unilateral removals of the anterior temporal cortex with 15 patients having unilateral removals of the amygdala and the hippocampus and 15 healthy controls. One experimental test given was of synonym judgments for 50 matched abstract and concrete words. Z-scores were derived from the performance of the healthy controls. The mean z-score for the anterior temporal patients was −1.06 for the abstract words but −3.53 for the concrete ones, significantly worse; by comparison the amygdala-hippocampal group scores virtually identically across the two types of word: −2.24 and −2.23, respectively. This supports the position that systems lying within the anterior temporal cortex are particularly important for processing the semantics of concrete by comparison with abstract words. This implies that the semantic processing of abstract words is in part dependent on other systems, a position originally put forward by Breedin et al. (1994) to explain the preservation of abstract word comprehension in their semantic dementia patient. Where might this other system be?

## Functional imaging studies

Functional imaging research has also led to the proposal that distinct systems may underlie the representations of abstract and concrete concepts (Binder et al., 2005). There is an extensive literature on neuroimaging studies of semantic processing (see Binder et al., 2009, for review). When processing of abstract words is contrasted with that of concrete words it tends to produce higher activation particularly in the left inferior frontal gyrus. Thus in a meta analysis of Wang et al. (2010), the left inferior frontal region was much the largest area that was consistently more activated for abstract than for concrete words.

However, the functional imaging evidence needs to be considered cautiously for a number of reasons. Firstly, many of the studies involve tasks, such as lexical decision, which make relatively small demands on semantic processing. This, however, means that the estimates of areas selectively involved in one or other type of semantic processing would be conservative. Two early studies that used lexical decision found somewhat surprising results. One using PET did find left inferior frontal gyrus to be more activated for abstract than concrete words (Perani et al., 1999) but many other regions in the right hemisphere were also involved. Kiehl et al. (1999) found only a right hemisphere region, namely the right superior temporal gyrus. However, neither used a random effects analysis and the Kiehl et al. study only had six subjects. Two later fMRI studies found effects much more limited to the left inferior frontal gyrus. In a study of Fiebach and Friederici (2004) only the left inferior frontal gyrus was involved, while in that of Binder et al. (2005) a somewhat larger left inferior frontal gyrus activation spread into the left precentral gyrus and to a small part of the left superior temporal gyrus.

One recent study did, however, not find any left inferior frontal gyrus activation when comparing abstract words with concrete ones, that of Vigliocco et al. (2013). The study was very impressive in that many nuisance variables were controlled between the abstract and concrete words sets. Altogether 14 variables were controlled including ones concerned with the orthography and phonology of the words, age and mode of acquisition, in addition to familiarity and frequency. However, another variable that was controlled was imageability. So the abstract word set included words such as *angel, demon, fury*, and *grief*, while the concrete words included ones like *product, relic, estate*, and *object* (Vigliocco, pers. commun.). Now neuropsychologically, where it has been examined in deep dyslexia, the key variable differentiating words easy and difficult for the patient to read was not concreteness *(C)* but imageability *(I)* (Shallice and Warrington, 1975). Thus for nouns relatively high in imageability or concreteness, 67% were read correctly by deep dyslexic patient, KF, if for the word *I* > *C*, but only 39% if *I* < *C* − 0.5^1^. The interpretation given at the time was that imagery was not itself the critical process, but whether the meaning of the word had been primarily learnt from visual experience. This is just the concept that was later used to explain what had been lost in semantic dementia patients showing a reversed concreteness effect (Breedin et al., 1994; Papagno et al., 2009a). Thus the Vigliocco et al. (2013) results are not relevant if one conceives of as abstract what cannot be learnt from sensory experience alone.

There is, however, a second problem with respect to the role of the left inferior frontal gyrus in activation by abstract concepts in lexical decision. The region is found to be activated in other lexical decision contrasts, in particular with low frequency words compared with high frequency ones, when concreteness is controlled (Fiebach et al., 2002). Thus, the region may be involved because of other processes, such as subvocalisation, especially as Fiebach et al. also found that in lexical decision pseudo words activated the region more than words (see also Fiebach et al., 2007). However, in the main Fiebach and Friederici (2004) study, reaction times to abstract and concrete words were virtually identical, so it is less plausible that additional mediation by subvocal rehearsal is occurring more for abstract words.

If, however, one moves to more demanding tasks, such as synonym judgments, there is yet another process, in addition to subvocalisation, which could be involved and which could lead to activation of left inferior frontal gyrus, namely working memory maintenance (Petrides, 1994). Intuitively, these two processes seem more likely to be involved in decisions on abstract words, as these tend to be the more difficult ones. Yet, when difficulty was specifically assessed in synonym judgment, it was not found to be the critical variable in the abstract-concrete contrast. Thus in the study of Noppeney and Price (2004), difficulty had a much weaker effect than abstraction *per se* on activation in the left inferior frontal gyrus. In the study of Sabsevitz et al. (2005) there was an area of overlap between difficulty and abstraction in left Brodmann area 45 but there were other parts of the left inferior frontal gyrus which were just activated by abstract rather than difficult concepts. Thus both with tasks making small demands on semantic processing and those making larger ones, the imaging findings are broadly consistent with the idea that a specific system in the left inferior frontal gyrus is involved in compiling the representations of abstract words.

A left inferior frontal gyrus localization also fits with inferences from other methodologies. The left inferior frontal lobe is a region which tends to be spared in semantic dementia prior to the later stages of the disease (Papagno et al., 2009a). Moreover in the three herpes encephalitis cases referred to above, the lesion appears not to extend to the left inferior frontal lobe; instead temporal cortices and the limbic system were held to be damaged. In none of the other cases of reversed concreteness effect reviewed by Papagno et al. (2009a,b) was the left frontal lobe held to be involved. A study using rTMS and lexical decision has also been carried out by Papagno et al. (2009b). They found that lexical decision to abstract words was less accurate after stimulation of the left inferior frontal gyrus instead of control sites, while no such effects were found for concrete words. A similar effect was also found for the left superior temporal gyrus. Overall, however, the inferior frontal gyrus appears to be critical for the semantic processing of abstract words.

## Contrasting properties of semantic representations of abstract and concrete terms

There is other neuropsychological evidence that the processing of abstract and concrete words differs qualitatively. This is shown in two studies of Crutch and Warrington (2005, 2007) on two patients. One patient, FBI, was a deep dyslexic. The other, AZ, had a semantic access/refractory disorder (see Warrington and Shallice, 1979; Warrington and McCarthy, 1987; Warrington and Cipolotti, 1996). Two types of similarity effects were examined to see if they differed between concrete and abstract words. The first was between semantically related members of a superordinate category, such as *yacht, dinghy, canoe, ferry*, and *barge* for concrete words or *fury, anger, rage, annoyance*, and *wrath* for abstract words. The contrasting situation was one in which the words differ in their superordinate semantic category but are linked by semantic association such as *dagger, blood, ambulance, policeman*, and *handcuffs* for concrete words or *democracy, republic, freedom, politics*, and *election* for abstract ones. For FBI two tasks were used: 4 and 5-alternative spoken word to written word matching and reading aloud the words in these sets. For each of the two types of word only one of the two kinds of similarity has a major effect, but it does so for both tasks. However, the other kind of similarity had little effect. For both tasks the critical effect for concrete words was belonging to the same category but for abstract words it was being within a group of associated words. Analogous findings were obtained with the two patients. This is evidence that the underlying semantic representations of concrete words and abstract words differ qualitatively not just quantitatively in their structure. Crutch and Ridgway (2012) prefer to see the semantics of the two types of word as both represented in a single distributed network. However, to us the contrasting semantic properties of the two types of word makes it at least as plausible that their semantic representations involve separable processing systems with different underlying micro-structure. To make this more plausible we need to consider how a semantic system or systems for concrete and abstract words might work computationally.

## The “hub” as a possible model of the semantic system

In order to consider whether the semantic representations of abstract and concrete concepts involve the same system or not, it is necessary to consider how each of them is composed. The computational model of the semantic system that provides currently the most plausible account of the semantic representations of concrete words is the “hub” model in which a central amodal semantic “hub” has a number of “spokes” representing different aspect of the concept (Rogers et al., 2004; Patterson et al., 2007). In the version of Jefferies and Lambon Ralph (2006) the spokes are verbal descriptors, visual, auditory, somatasensory and olfactory/gustatory features and “praxis”. The hub learns to transform input corresponding to one aspect of a concept derived from one of the spokes to produce an output to a different spoke, corresponding to another aspect. The concepts and features used to train the Rogers et al. net are derived from a study of Garrard et al. (2001). If we leave on one side superordinate concepts, then the typical more dominant features of the 32 living thing and 32 artifact concepts Garrard et al. studied are indeed codeable in representations in one of these spoke systems e.g., *visual—alligator: has tail, barrel: is made of wood; auditory—aeroplane: can make a noise, dog: can bark; somatosensory—axe: is sharp, cat: can scratch; olfactory/gustatory—apple: is sweet, pineapple: is juicy; praxis—basket: can be filled, bicycle: can be ridden.*

The hub model is not without its internal difficulties. In particular it is unclear how one form of category specificity—the superior performance with artifact knowledge compared with living thing knowledge quite frequently reported in herpes simplex encephalitis patients—can occur with very similar lesion sites to semantic dementia where such category specificity is very rare (Garrard et al., 1998; see Lambon Ralph et al., 2007; Shallice and Cooper, 2011, for discussion). However, we consider it a plausible model of the semantics of concrete words as it can account for many striking phenomena with respect to semantic dementia itself (Patterson et al., 2007).

## Conceptual limitations of the hub model with respect to abstract words

In the hub model a concrete concept like sparrow is represented by a list of features: *isa bird, is small, has wings, is brown, chirps*, etc. To a non-expert the presence or absence of features are apparently independent of each other; that a sparrow is small, drab and chirps and a parrot is larger, highly colored and squawks appear to be just two possibilities in a three dimensional space where any of eight possibilities are equally likely. But what does a feature mean? The features listed above have two parts—what one might call an operator e.g., *potential action (can be …)*, and an argument e.g., *filled* or *ridden* (as in *basket: can be filled*, or *bicycle: can be ridden*). Thus, within the hub model the content of a concept such as bicycle may be represented in more formal terms as a conjunction of features with each feature comprising an operator and an argument:

(1) $$\begin{array}{l} Bicycle(X)\text{ if and only if }isa(X,vehicle)\text{ AND}\\ \;has(X,seat)\text{ AND}\\ \;has(X,wheels)\text{ AND}\\ \;canbe(X,ridden)\text{ AND}\\ \;\ldots \end{array}$$

In the features given above the operator is specified by the spoke subsystem so in the case of *bicycle: can be ridden* it derives from the spoke system being the praxis one. The set of operators available is therefore limited by the set of spoke systems and these are highly restricted in number even if one considers subcategories of feature; for vision, examples would be the operators *has a X* or *made of X* derived from object-form or texture representations, respectively. Thus on the hub model a concrete concept has a list structure of features and the operator part of an individual feature is specified by the specific spoke that activates the feature.

Consider instead an abstract concept like *tendency* or *hope. Tendency* does have visual or spatial aspects, such as a 10° angled line approaching the horizontal, but they are few in number, far from being distinctive to *tendency* and cannot without additional information specify the concept. *Hope* too has visual aspects, such as a generally positive expression but they are as little distinctive to *hope* as a 10° line is to *tendency*, and distinctiveness is a key property for learning a concept^2^. Moreover unlike a concrete concept, their core semantic representation is not well captured by a list of independent features with access to the representation requiring that only a subset of the full list of features be activated. Instead, the concepts *tendency*, in its WordNet (Fellbaum, 1998) sense of *“a characteristic likelihood of or natural disposition toward a certain condition or character or effect”*, and *hope*, in its WordNet sense of *“to intend with some possibility of fulfilment”*, need to be captured by something equivalent to:

(2) Tendency(X) if and only if 1.0>probability(X)>chance

(3) Hope(X) if and only if desire(X)AND believe(possible(X))

For such concepts a much wider set of operators like *desire*, *believe*, and *possible*, as well as representational abilities related to *probability* are required. Even more critically, the logical relations between the different elements of the whole representation are much more complex than the simple list structure that, say, the hub model provides. This is both reflected in the recursive embedding of operators (e.g., *believe (possible (X))*) and by the fact that the *X* in (2) and (3) is an event or state of the world, in contrast to (1) where it corresponds to a physical object. Moreover, in the former cases the state of the world referred to by *X* is not the current or actual state of the world but a hypothetical, possible state of the world.

What specific representational abilities might be required for these concepts? Within the fields of mathematical logic and formal semantics, providing an account of the meaning of statements such as “it is possible that *X*” led to the development by Lewis (see Lewis and Langford, 1932) of so-called “modal logics” (specifically logics of necessity and possibility, and logics of knowledge and belief) and in particular to the development by Kripke (1959) and others of “possible world semantics”. The central idea behind semantic theories of this general kind is that the meaning of a statement *X* is determined with respect to a model or “world”. Modal logics augment traditional predicate logic with modal operators such as *necessary* and *possible*, or *know* and *believe*, while possible world semantics provides a semantic theory in which the meaning of these operators is provided via the abstract concept of a “possible world”.

A possible world may be thought of as a set of atomic tokens and relations between those tokens where the relations are internally consistent. Thus, if the possible world includes a relation such as *larger-than* then this relation must be transitive within the possible world. Tokens may correspond to concrete objects in the real world or to abstract entities (such as “a job”). Informally a possible world can be thought of as similar to a mental model (Johnson-Laird, 1983) [see in particular, Perner (1988) for discussion of possible world semantics in the representation of mental states]. Formally, the requirement of internal consistency means that a possible world is closed with respect to the deductions that it supports. Thus, if *A* is true in a world *W* and *A* implies *B* then *B* must also be true in *W*. A statement of the form *possible (X)* is true if and only if there exists at least one possible world in which *X* is true, while *necessary (X)* is true if and only if *X* is true in all possible worlds^3^. Critically, while the core meaning of *bicycle* as in definition (Equation 1) can plausibly be provided as a set of features or within predicate logic (with a standard so-called extensional semantics), the meaning of *tendency* and *hope* cannot—additional machinery such as modal logic and possible world semantics is required^4^.

We are not suggesting that all abstract words require modal logic in order to adequately characterize their core meaning, or that modal logic alone can capture the core meaning of all abstract words. Rather, the claim is that the meanings of abstract words cannot be adequately captured purely in terms of a list of perceptually grounded features, as provided by the hub model. As a further example, consider *democracy*, which is defined in WordNet as “a political system in which the supreme power lies in a body of citizens who can elect people to represent them”. This sense of *democracy* is not readily characterizable either as a set of perceptually grounded features or as a proposition in a modal logic. At the very least it is related to concepts of *statehood*, *government* and *election* in a way that is qualitatively different from the relation between, for example, *bicycle* and *wheels*.

## The separable systems approach

In Shallice and Cooper (2011) we argued that differences from concrete concepts in the computational requirements for how they are represented in an underlying semantic system, such as those discussed above, make it plausible that representing the meanings of abstract concepts involves a different computational system than that involved in representing the meanings of concrete concepts. Functionally, this system would need to incorporate the ability to abstract over events or situations rather than just individuals, to apply modal operators recursively, and to allow the representation of hypothetical as well as actual events or situations. Moreover if we consider how the representation of an event might be realized computationally, then the binding of argument roles to arguments is required (see Shastri, 2002); thus representing an event like the *giving of a gift* requires filling the roles of the gift *giver*, the gift *recipient* and the gift *object*.

We should make two qualifications to this position. The first qualification is that the evidence we have reviewed does not distinguish between two possibilities. One is that the left inferior frontal gyrus is the location of the semantic representations of abstract words. The second is that it is critically involved in processes necessary to access or construct these representations.

Secondly, we presume that one type of representation of an event, including binding, can take place in parieto-temporal systems, namely perceptual representations of the current world or of a sensory (e.g., visual) image, loosely what at the psychological level (with premotor systems) is we assume to be carried by the concept *embodied cognition*. However, using Shastri's example, what is represented at this level of processing is person *A* handing a concrete object (e.g., a book) to person *B*. What is not represented is that the object is a *gift*, and all the many culturally dependent implications this has for the giver and the recipient. Thus even though parieto-temporal systems can capture the representation that a particular glittering object is gold (or not as the case might be!), impairments in understanding the abstract meaning of a proverb such as *all that glitters is not gold*—that appearance does not necessarily correspond to essence—instead involves prefrontal cortex (Murphy et al., submitted). In particular, left lateral patients produce more than four times more concrete interpretations of such proverbs than do healthy controls. It is compatible with their lacking such representations. For representations at such higher non-perceptual levels, binding would, we assume, not be available in parietal cortex.

At the very least an abstract concept semantic system would need the power to implement recursion and argument role filling, neither of which is, for instance, available in the architecture of the hub system. We further argued in Shallice and Cooper (2011) that given requirements such as these, it would be plausible that the computational microstructure of the region of the human cortex supporting the representation and processing of abstract concepts would be different from that of the anterior temporal cortex held to support the representation of concrete concepts, and proposed on the basis of functional imaging and patient studies that this abstract representational system was located in the left ventrolateral prefrontal cortex.

In the psychological literature, the idea that word meaning involves more than just a list of semantic features is, of course, old. Indeed, Miller and Johnson-Laird (1976) argue that the meanings are represented by mini-programs. Thus they represent the meaning of *LOSE(x, w)* by^5^:

*Someone x loses something w if there is a time t such that Q*_*t*_*(POSSESS(x, w)) and:*

- *R*_*t*_*(notINTEND(x, notPOSSESS(x, w)))*
- *HAPPEN*_*t*_*(NotPOSSESS(x, w))*

Miller and Johnson-Laird (1976, p. 568)

The basic difference in our position from this earlier perspective is that in our view such program-like entities are critical for representing the meaning of abstract words, but while they coexist with feature-based ones, they are in a functionally and anatomically separable system.

The role that we have assigned to the left inferior frontal gyrus has another if slightly more indirect precursor. The computational machinery which we have proposed for this region with respect to the compiling of the meaning of abstract words has many similarities to that presupposed for compiling syntax. In particular our position on abstract words, too, requires that unification links be made between the arguments of two or more operators, as in the example of *hope* above (see, e.g., Pollard and Sag, 1987, 1994, for unification in syntactic operations). Hagoort (2003), too, has argued that the left inferior frontal gyrus contains the necessary computational machinery for implementing unification processes. In his account chunks of syntactic structure (e.g., S, NP, VP, N, and V) of an utterance are stored in memory. In a unification workspace the feet of one syntactic chunk are potentially linked to the root of another. In the computational model of Vosse and Kempen (2000), which he adopts, rival sets of unification links for spanning a whole utterance (e.g., a sentence) compete by lateral inhibition until one reaches threshold. In Hagoort's account this process of forming provisional sets of links which compete by lateral inhibition takes place in the left inferior frontal gyrus. In later papers (e.g., Hagoort, 2005) he extends this idea to consider semantics, with semantic unification being held to take place in a region a little more inferior and anterior than that for syntactic unification. The form of semantic unification he considers is the integration of word meaning into an unfolding discourse representation of the preceding context, for instance in the selection of the appropriate meaning of a homonym. Our proposal is that an analogous process may underlie the semantic representations of individual abstract words.

Of course it may be argued that *unification* as a concept is little more than *binding* which is widely postulated to occur in many cognitive processes, as in episodic memory encoding in the hippocampus (Marr, 1971; Gardner-Medwin, 1976) or perceptual feature-binding in parietal cortex (Treisman, 1998). The critical formal difference between unification and binding is that the former combines multiple potentially overlapping sources of information. Unification will fail if overlapping elements of the to-be-combined representations are inconsistent. Moreover unification is typically used in building complex structures (e.g., where multiple arguments serve different functional roles) out of parts, and where the parts place constraints on each other. Thus what we assume distinguishes the unification process taking place in left inferior frontal cortex is that the item or element is being bound to a node within a more complex structure representing an abstract general property such as *propositional phrase* or *type of mental state*.

How does this position relate to the cognitive neuroscience evidence just discussed? If the computational properties of an abstract concept semantic system were designed in part to allow events to be represented, Crutch and Warrington's findings that associations are critical in the representations of abstract words would seem to follow. A set of words like *gamble, casino, poker*, and *chance*, ones used in Crutch and Warrington's (2005) experiment on interference from associated sets, almost inevitably creates a characteristic situation or set of events related to playing poker, as does the example *democracy, republic, freedom, politics*, and *election* discussed earlier, redolent of the 2012 American election; so the individual abstract semantic representations would be linked to each other through it.

A second phenomenon which has been held to support the idea that the semantics of abstract words can be represented in the hub and hence to present difficulties for an abstract semantic system account comes from a rTMS study of Hoffman et al. (2010). They followed Schwanenflugel and Shoben (1983) in assuming that the same semantic system is involved for abstract and concrete words but the precise meaning of an abstract concept is heavily dependent on context. (It is not clear whether or how this would apply to concepts like *neutron* or *checkmate*.) They gave subjects a 3-alternative synonym judgment task together with a target word presented altogether at the same time or also preceded for 6 s by a 2-sentence context. Without the context, slower responding to abstract words occurred with rTMS to left Brodmann area 45 than without it. However, with context no such effect occurred. With concrete words, rTMS has no effect in either case.

Hoffman et al. (2010) explain their result as occurring through left ventrolateral prefrontal cortex having an executive regulation role with respect to the processing of abstract words, and this becomes less necessary when context is provided. It is not, though, clear, what computational function executive regulation plays in understanding an abstract word in a context-free situation. Moreover there are possible alternative explanations of the result. rTMS does not lead to any increase in errors with abstract words, it just leads to slowing in the no-context condition. Thus in the context situation the subject will have already understood the word, which has already been presented in the context, at least in the example given, so the subject will just have to comprehend one critical word instead of two, and so at worst will presumably be slowed up only half as much. As the no-context effect was only just significant at the 0.05 level one would not therefore predict a significant effect in the context case even if the left ventrolateral PFC was as critical there. Moreover, even if full abstract comprehension of the three choice words is slowed, the 6 s of context presentation will have left a rich set of concrete images from parieto-temporal regions available to facilitate the choice between the three alternatives, at least on some trials, so this again would be expected to reduce any effect in the context condition compared to the no-context one. The study, by itself does not resolve the issue.

## Conclusions

Our primary conclusion is a negative one. It is that the computational capacities provided by embodied cognition, on the one hand, and the feature-based representation of semantics on other hand (and more specifically the “hub” system), are insufficiently powerful to adequately capture the semantics of abstract concepts. Moreover we have argued that patients with reversed concreteness effects on the one hand and deep dyslexia on the other provide some evidence that the semantic representations of abstract and concrete words are at least partially separable in the cognitive system. This position is further supported by the different patterns of interference and facilitation found by Crutch and Warrington in their single case studies. Neuroimaging evidence, too, suggests that the left inferior frontal cortex plays a more important role in the compiling of the semantics of abstract than concrete words.

The computational characteristics that we have ascribed to an abstract representational system have a very similar conceptual basis to—but are different from—those involved in grammatical/syntactic operations in language. If the microstructure of cortex is critical for the computational properties of the functional systems it supports, then it is plausible that systems with similar computational requirements are supported by overlapping or adjacent regions of cortex. It is therefore not surprising that a similar region of cortex would be involved in the representation of abstract concepts to that damaged in agrammatism (e.g., Tyler et al., 2005, 2010, 2011). Moreover deduction, which also requires similar computational properties in the construction of abstract structures in premise integration, also involves a very similar region (Reverberi et al., 2012). Two qualifications should be made. The first is that the operational definitions of abstraction used in empirical studies have typically been made apophatically or negatively, by the absence of concreteness in the entity, or as we have argued neuropsychologically more appropriately, the absence of imageability of the concept. Ideally on our approach, one ought to be able to produce an operationalization of abstraction which is positive rather than negative. Until this is done, direct empirical support for a position such as ours will be difficult to obtain.

The second qualification relates to the way that the general thrust of this paper may be interpreted as suggesting that representations of abstract concepts are held in the left inferior frontal gyrus. Moreover, the link we have made between our approach and Hagoort's unification concept tends to reinforce that view. However, the direct empirical cognitive neuroscience evidence is open to a second interpretation. This is that the representations of abstract concepts are carried in a more distributed fashion, possibly more generally in prefrontal cortex. In this case the left inferior frontal region would be crucial in performing appropriate computations to compile the more distributed representations. Which of these two possibilities is to be preferred empirically remains in our view an open question. In either case, though, there would be more to the mind than embodied cognition.

### Conflict of interest statement

The authors declare that the research was conducted in the absence of any commercial or financial relationships that could be construed as a potential conflict of interest.

## Footnotes

^1^*I* and *C* were as normed by Paivio et al. (1968): compare journal (*I* = 5.60; *C* = 6.69) versus winter (*I* = 6.53; *C* = 5.83).

^2^The hub is also linked to “Executive control” in the Jefferies and Lambon Ralph (2006) version but this is held to “help direct and control semantic activation in a task-appropriate fashion” (p. 2132). It does not provide comparably functioning input to the other “spoke” systems. Thus few if any features seem to be located there. In any case, neither *tendency* nor *hope* seem to have executive control aspects.

^3^Possible world semantics normally also includes an “accessibility” relation, such that *possible(X)* is true if and only if *X* is true in some world accessible from the current world, while *necessary(X)* is true if and only if *X* is true in all worlds accessible from the current world. For simplicity we ignore this relation in the current discussion.

^4^Van Bentham (1976) demonstrated that there is an equivalence between some modal logics and first-order predicate logic which can be obtained by mapping a statement *P(x)* in modal logic to the statement *P'(w, x)* in first order logic where *w* is the current world, and allowing quantification over possible worlds, so that, for example, *possible (P(x))* becomes ∃w *P' (w, x)*. While this demonstrates that modal logic per se is not required to provide a semantics for words such as *hope*, it does not obviate the need to quantify over possible worlds or hypothetical states in providing that semantics.

^5^*Q*_*t*_ and *R*_*t*_ are operators within a temporal modal logic. *Q*_*t*_(*P*) is true if *P* was true prior to time *t* and *R*_*t*_(*P*) is true if *P* is true at time *t*. *Q*_*t*_ might be glossed as “it was the case that…” and *R*_*t*_ as “it is the case that…”.
